# Admission body temperature and in-hospital mortality in critically ill children with pneumonia: a retrospective cohort study

**DOI:** 10.1186/s12887-026-07300-9

**Published:** 2026-07-10

**Authors:** Xiaojuan Zhang, Xiaofang Ma, Wei Wang, Liping Liu, Jie Chang

**Affiliations:** 1 Department of Respiratory Medicine, Shanxi Provincial Children’s Hospital, Taiyuan, China; 2 Department of Pediatrics, Taiyuan Maternity and Child Care Hospital, Taiyuan, China

**Keywords:** Pneumonia, Body temperature, Hyperthermia, Pediatric intensive care unit, Mortality

## Abstract

**Objective:**

To evaluate whether admission body temperature (BT) is associated with in-hospital mortality in critically ill children with pneumonia.

**Design:**

Retrospective cohort study.

**Methods:**

This study employed a retrospective cohort design using records derived from the Pediatric Intensive Care (PIC) database collected between 2010 and 2018. Children aged > 28 days and < 18 years who were admitted to the PICU with a diagnosis of pneumonia were included in the analysis. Based on BT measured at admission, patients were grouped as hypothermic (< 36.5℃), normothermic (36.5–37.4℃), or hyperthermic (≥ 37.5℃), with additional classification according to the degree of hyperthermia. The association between admission BT and in-hospital mortality was evaluated using multivariable logistic regression. Furthermore, restricted cubic spline modeling, along with subgroup and sensitivity analyses, was undertaken to test the consistency of the findings.

**Results:**

In total, 394 pediatric patients with severe pneumonia were retained for analysis. Admission BT was independently linked to in-hospital mortality, with higher values corresponding to an increased risk of death. When compared with patients presenting with normal BT, those with hyperthermia exhibited a substantially greater likelihood of mortality (OR 3.10, 95% CI 1.26–7.62), and this risk was even more pronounced among individuals with severe hyperthermia (OR 5.74, 95% CI 1.55–21.20). Hypothermia, however, showed no statistically significant association with mortality. Analysis using restricted cubic splines supported a linear pattern between BT and mortality risk. Subgroup analyses revealed no significant effect modification by age, gender, or SpO2.

**Conclusions:**

Among critically ill children with pneumonia, elevated admission BT—particularly severe hyperthermia—was associated with increased in-hospital mortality. Admission BT may be a readily accessible and clinically informative parameter for early risk stratification in the PICU.

**Clinical trial number:**

Not applicable.

**Supplementary Information:**

The online version contains supplementary material available at https://doi.org/10.1186/s12887-026-07300-9.

## Introduction

Pneumonia remains a major contributor to childhood mortality worldwide, with a particularly pronounced impact among younger children and those with critical illness [1]. Although substantial advances have occurred in antimicrobial therapy and pediatric critical care over recent decades, mortality from severe pediatric pneumonia remains non-negligible, and clinical outcomes vary considerably across individuals. Epidemiological evidence drawn from both global and national datasets indicates that pneumonia consistently ranks among the leading causes of death in children, particularly in vulnerable populations and resource-constrained settings [2–4].

Within the pediatric intensive care unit (PICU), children are typically admitted with severe or complex illness, highlighting the need to promptly identify patients at increased risk of adverse outcomes and to implement timely and objective risk stratification strategies.

Body temperature (BT) is a routinely recorded physiological parameter in clinical practice. Abnormal BT reflects the physiological response of the host to infectious or inflammatory stimuli and is therefore widely incorporated into disease severity assessment frameworks [5–9]. However, the prognostic significance of BT is not uniform across clinical contexts and levels of disease severity, particularly in critically ill populations, and remains incompletely defined [10–13]. In pediatric populations, existing evidence has primarily focused on neonates and children with sepsis [12, 14, 15], whereas data specific to critically ill children with pneumonia admitted to the PICU remain scarce, especially regarding systematic evaluation of the association between admission BT and mortality outcomes.

In our previous study involving a heterogeneous population of critically ill children admitted to the PICU, we investigated the association between admission BT and 28-day mortality. Subgroup analyses demonstrated a significant interaction between BT and pneumonia status (P for interaction < 0.001). Specifically, elevated body temperature was associated with a markedly increased risk of mortality among children with pneumonia (HR 4.24, 95% CI 2.59–6.94), suggesting that pneumonia may modify the association between body temperature and mortality [13]. However, evidence specifically focusing on critically ill children with pneumonia remains limited.

Based on this background, this study conducted a retrospective cohort analysis using data from the Pediatric Intensive Care (PIC) database to examine the association between admission BT and in-hospital mortality in critically ill children with pneumonia. In addition, we evaluated the potential value of admission body temperature for early risk stratification in this population.

## Materials and methods

### Data source

This study used data derived from the PIC database, a large, publicly available pediatric critical care repository established by the Children’s Hospital, Zhejiang University School of Medicine, which includes de-identified medical records from 12,881 pediatric patients accounting for 13,944 ICU admissions between 2010 and 2018 [16]. The database covers demographic characteristics, vital signs, laboratory measurements, and clinical outcomes. All records were anonymized to ensure patient privacy. Ethical approval was obtained from the Institutional Review Board of the Children’s Hospital, Zhejiang University (Approval No. 2019-IRB-052), and the requirement for individual informed consent was waived because of the retrospective nature of the study and the use of anonymized data.

### Study design and participants

**Fig. 1:**
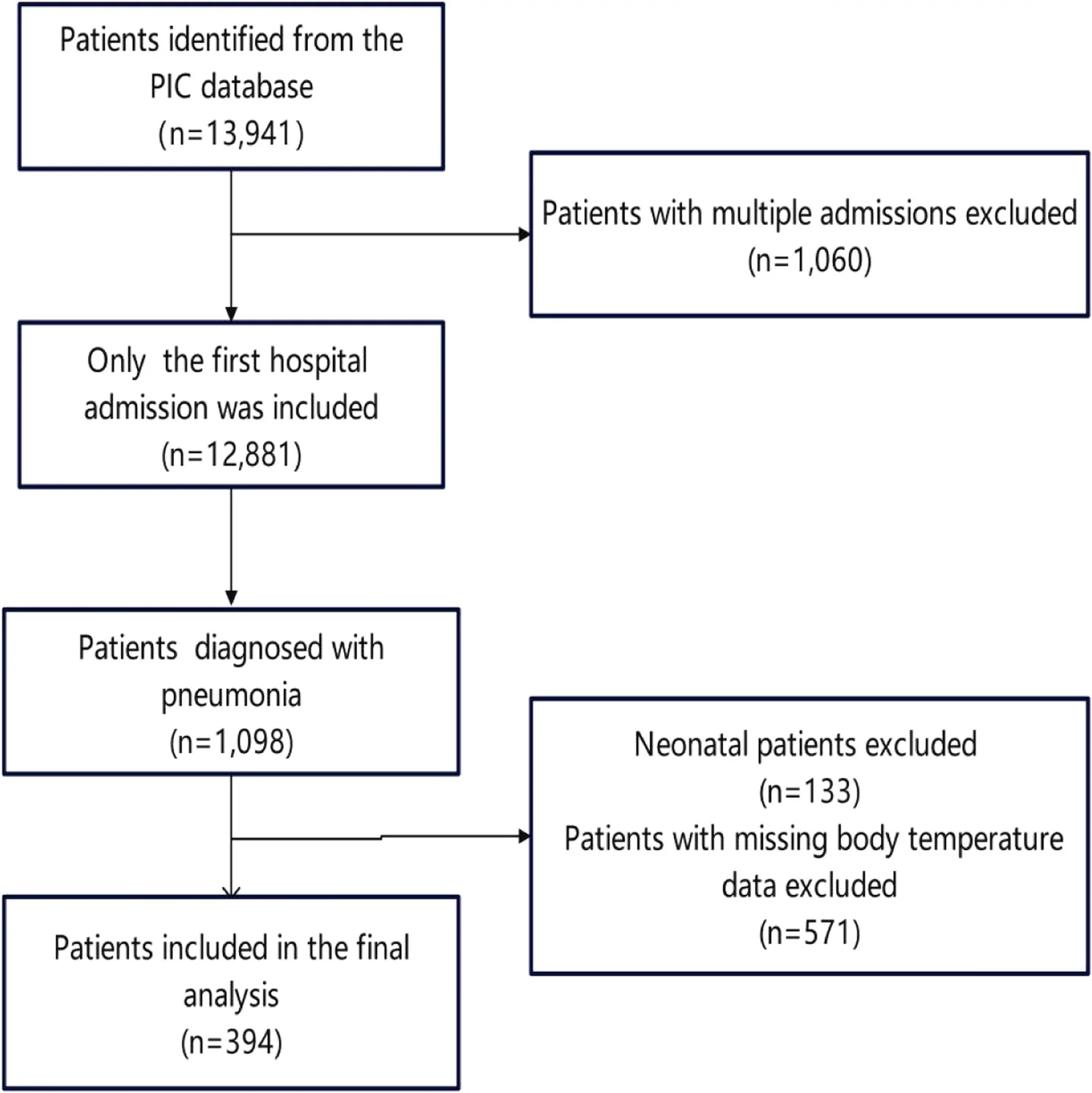
Flowchart of patient selection

A retrospective cohort framework was applied to identify pediatric patients aged > 28 days and < 18 years who were admitted to the PICU with a diagnosis of pneumonia, as defined by International Classification of Diseases, 10th Revision (ICD-10) codes. In cases of repeated hospitalizations, only the earliest admission was considered for analysis. Tympanic temperature measurements obtained within 24 h of PICU entry were extracted, and the first recorded tympanic temperature was defined as the admission BT. Records without available BT data were excluded. Following implementation of the predefined eligibility criteria, a total of 394 critically ill children with pneumonia were included in the final analytical cohort (Fig. 1). Based on previously published studies in critically ill pediatric populations, patients were stratified according to admission BT into hypothermia (BT < 36.5 °C), normothermia (36.5 °C ≤ BT < 37.5 °C), and hyperthermia (BT ≥ 37.5 °C) groups [12, 13]. To allow a more detailed evaluation of the relationship between elevated BT and mortality, the hyperthermia category was further classified as mild (37.5 °C ≤ BT ≤ 38.0 °C) or severe (BT > 38.0 °C).

### Data extraction and outcome definition

Relevant study variables were retrieved from the PIC database using structured query language (SQL). Collected information included demographic data (age and gender), vital signs first documented within 24 h after PICU admission–including BT, average temperature, respiratory rate, heart rate, and peripheral oxygen saturation (SpO₂)–as well as laboratory parameters measured during the same 24-hour period. These laboratory indices included white blood cell count (WBC), neutrophil count (NEUT), platelet count (PLT), hemoglobin (HGB), C-reactive protein (CRP), serum albumin (ALB), alanine aminotransferase (ALT), glucose (GLU), lactate, pH, pCO_2_, potassium (K), calcium (Ca), and phosphate (P). Data on ICU length of stay and patient survival status were additionally obtained.

BT was defined as the first documented temperature measurement within 24 h after PICU admission. The average BT on the first day was calculated as the arithmetic average of all temperature measurements recorded during the first 24 h. The primary study outcome was in-hospital mortality, defined as death occurring during the index hospitalization, whereas patients discharged alive were classified as survivors.

### Statistical analysis

Baseline data were described using appropriate summary statistics. Categorical variables were expressed as frequencies and percentages, while continuous variables were presented as means with standard deviations or medians with interquartile ranges, according to data distribution. Comparisons among BT categories were performed using Pearson’s chi-square test for categorical variables, one-way analysis of variance for normally distributed continuous variables, and the Kruskal–Wallis test for non-normality distributed variables.

The association between BT and in-hospital mortality was examined using multivariable logistic regression models. Effect estimates are reported as odds ratios (ORs) with 95% confidence intervals (CIs). BT was modeled both as a continuous variable (per 1 °C increase) and as a categorical exposure (hypothermia, normothermia, and hyperthermia), with normothermia serving as the reference category. Candidate covariates were considered for model inclusion based on univariate screening (*P* < 0.05), established clinical relevance, or a change-in-estimate criterion of > 10%. Sequential models were fitted to assess the stability of the associations: Model I, unadjusted; Model II, adjusted for age and gender; Model III, further adjusted for respiratory rate and SpO_2_; Model IV, additionally adjusted for WBC, CRP, GLU, and lactate.

Potential non-linear relationships between BT and in-hospital mortality were explored using restricted cubic spline (RCS) models based on the fully adjusted Model IV. Evidence of non-linearity was evaluated by comparing the spline-based model with a corresponding model that included only a linear term.

To determine whether the association between BT and in-hospital mortality differed across patient subgroups, predefined subgroup analyses were conducted according to gender, age, and SpO_2_ categories. Effect modification was evaluated by including interaction terms in logistic regression models, and interaction terms were tested with likelihood ratio tests to assess statistical significance.

In all analyses, the proportion of missing values among covariates was less than 10%. Assuming that missing covariate data were missing at random (MAR), missing values were handled using multiple imputation with five datasets to reduce potential bias and preserve statistical power. To test the robustness of the main findings, we performed a series of complementary analyses, including repeating the primary models with the average BT measured during the first 24 h after PICU admission, restricting analyses to complete cases, further adjusting for vasoactive drug use, evaluating baseline differences between patients with complete and incomplete covariate data, and comparing baseline characteristics between patients with and without available admission BT data.

Statistical analyses were carried out using R software (version 4.4.1; R Foundation for Statistical Computing, Vienna, Austria) and Free Statistics software (version 2.2; Beijing FreeClinical Medical Technology Co., Ltd., Beijing, China). All hypothesis tests were two-sided, and statistical significance was defined as a *P* < 0.05.

## Results

### The selection of participants

From the PIC database, a total of 13,941 pediatric ICU admissions were screened. After excluding 1,060 repeated admissions, 12,881 unique first ICU admissions were retained. Among these, 1,098 patients with pneumonia were identified based on ICD-10 codes. After excluding 133 neonates and 571 patients with missing BT data, a final cohort of 394 critically ill children with pneumonia was included in the analysis (Fig. 1).

### Study participants and baseline characteristics

Overall, 394 pediatric patients diagnosed with pneumonia were included in the analysis. The cohort comprised 99 (25.1%) patients with hypothermia, 184 (46.7%) with normothermia, and 111 (28.2%) with hyperthermia. Baseline characteristics stratified by BT category are shown in Table 1.

**Table 1:** Clinical characteristics of the study population

Variable	Total	Hypothermia (BT < 36.5 °C)	Normothermia (36.5 ≤ BT < 37.5 °C)	Hyperthermia (BT ≥ 37.5 °C)	*P* value
N	394	99	184	111	
Gender (male, %)	224 (56.9)	54 (54.5)	105 (57.1)	65 (58.6)	0.839
Age(months)	6.8 (2.9, 22.2)	4.9 (2.4, 10.8)	4.9 (2.4, 22.5)	14.2 (6.3, 42.9)	< 0.001
Los hospital(day)	16.0 (8.0, 27.0)	19.0 (10.5, 30.5)	15.5 (9.8, 28.2)	14.0 (6.0, 22.0)	0.009
Los ICU (day)	5.0 (2.0, 12.0)	3.0 (1.0, 9.0)	5.0 (2.0, 15.0)	5.0 (3.0, 12.5)	0.015
In-hospital mortality, n (%)	36 (9.1)	4 (4)	12 (6.5)	20 (18)	< 0.001
Vital signs					
BT (℃)	37.0 ± 0.9	36.0 ± 0.4	36.9 ± 0.3	38.1 ± 0.7	< 0.001
Average temperature (℃)	37.0 ± 0.8	36.3 ± 0.5	37.0 ± 0.4	37.8 ± 0.7	< 0.001
Respiratory rate (beats/min)	39.3 ± 12.3	35.1 ± 10.1	39.6 ± 11.6	42.5 ± 14.2	< 0.001
Heart rate (beats/min)	138.1 ± 26.4	133.2 ± 23.3	138.3 ± 24.4	142.4 ± 31.4	0.051
SpO₂ (%)	97.3 ± 3.0	97.9 ± 2.2	97.1 ± 3.5	97.0 ± 2.6	0.122
Laboratory data					
WBC (×10^9^/L)	9.0 (5.9, 13.9)	7.9 (6.3, 11.4)	9.5 (5.8, 14.1)	9.8 (6.0, 15.8)	0.136
NEUT (×10^9^/L)	5.0 (2.9, 9.6)	4.7 (2.7, 8.0)	4.8 (2.6, 9.1)	6.7 (3.4, 11.3)	0.033
PLT (×10^9^/L)	311.7 ± 168.2	304.0 ± 160.9	327.4 ± 175.5	292.1 ± 161.1	0.203
HGB(g/L)	104.7 ± 18.8	100.0 ± 19.3	106.0 ± 19.1	106.7 ± 17.3	0.018
CRP (mg/L)	5.0 (2.0, 22.6)	5.0 (2.0, 17.0)	4.0 (1.0, 12.8)	10.0 (3.0, 38.0)	0.008
ALB (g/L)	37.2 ± 6.1	37.4 ± 5.7	36.9 ± 5.8	37.7 ± 6.8	0.613
ALT (U/L)	25.0 (17.0, 40.0)	26.0 (16.0, 39.0)	23.0 (17.0, 35.8)	26.0 (16.8, 45.2)	0.700
LDH (U/L)	396.0 (298.5, 565.0)	359.0 (285.5, 477.0)	391.0 (291.2, 574.0)	462.0 (337.2, 666.5)	0.002
GLU (mmol/L)	7.5 ± 3.0	7.5 ± 3.1	7.5 ± 2.8	7.5 ± 3.2	0.994
Lactate (mmol/L)	1.7 (1.2, 2.5)	1.6 (1.1, 2.3)	1.6 (1.1, 2.5)	1.9 (1.3, 2.5)	0.366
pH	7.4 ± 0.1	7.4 ± 0.1	7.4 ± 0.1	7.4 ± 0.1	0.174
pCO2 (mmHg)	45.6 ± 16.3	43.3 ± 12.2	47.3 ± 17.1	44.7 ± 18.0	0.116
K (mmol/L)	4.0 ± 0.8	3.9 ± 0.7	4.1 ± 1.0	3.9 ± 0.6	0.168
Ca (mmol/L)	1.2 ± 0.1	1.2 ± 0.1	1.2 ± 0.1	1.1 ± 0.1	< 0.001
P (mmol/L)	1.6 ± 0.5	1.6 ± 0.4	1.7 ± 0.5	1.5 ± 0.5	0.061
Vasoactive drug, n (n%)					0.019
No	386 (98.0)	94 (94.9)	181 (98.4)	111 (100)	
Yes	8 ( 2.0)	5 (5.1)	3 (1.6)	0 (0)	

*Abbreviations*: *SpO*₂ Peripheral oxygen saturation, *WBC* White blood cell count, *NEUT* Neutrophil count, *PLT* Platelet count, *HGB* Hemoglobin, *CRP* C-reactive protein, *ALB* Albumin, *ALT* Alanine aminotransferase, *LDH* Lactate dehydrogenase, *GLU* Glucose, *pCO*_2_ Partial pressure of carbon dioxide, *K* Potassium, *Ca* Calcium, *P* Phosphate Continuous variables are described as mean ± SD or median (IQR) according to the normality of distribution

Significant between-group differences were observed in age, hospital length of stay, ICU length of stay, and in-hospital mortality (all *P* < 0.05). Patients assigned to the hyperthermia group were older and experienced the highest in-hospital mortality when compared with those in the normothermia and hypothermia groups. Gender distribution and SpO₂ were comparable across the three groups. With regard to vital signs, both admission BT and average BT varied significantly across temperature categories, and additional differences were noted in respiratory rate and heart rate. In laboratory assessments, statistically significant variations were observed for NEUT, HGB, CRP, LDH, and Ca among groups. In addition, the proportion of patients receiving vasoactive drugs differed significantly across BT categories. In contrast, no significant differences were found for WBC, PLT, ALB, GLU, lactate, K, or P across BT categories (all *P* > 0.05).

### Association between BT and clinical outcomes

Results from both univariate and multivariable analyses (Supplementary Table S1 and Table 2) indicated a significant association between BT and in-hospital mortality. With normothermia serving as the reference category, hypothermia showed no significant relationship with in-hospital death across all fitted models. Conversely, hyperthermia was significantly associated with increased mortality risk across all models. The estimated odds ratios for hyperthermia were 3.15 (95% CI 1.47–6.73) in Model I, 3.24 (95% CI 1.50–6.99) in Model II, 2.51 (95% CI 1.06–5.94) in Model III, and 3.10 (95% CI 1.26–7.62) in Model IV.

**Table 2:** ORs for in-hospital mortality with three levels of body temperature

Variable	Model Ⅰ		Mode Ⅱ		Model Ⅲ		Model Ⅳ	
	OR (95%CI)	*P* value	OR (95%CI)	*P* value	OR (95%CI)	*P* value	OR (95%CI)	*P* value
Temperature (*n* = 394)	2.18 (1.52 ~ 3.13)	< 0.001	2.22 (1.54 ~ 3.2)	< 0.001	1.79 (1.19 ~ 2.69)	0.005	2 (1.29 ~ 3.09)	0.002
Hypothermia (*n* = 99)	0.6 (0.19 ~ 1.92)	0.394	0.6 (0.19 ~ 1.9)	0.381	0.97 (0.28 ~ 3.28)	0.957	0.95 (0.27 ~ 3.34)	0.931
Normothermia (*n* = 184)	1 (Ref)		1(Ref)		1(Ref)		1(Ref)	
Hyperthermia (*n* = 111)	3.15 (1.47 ~ 6.73)	0.003	3.24 (1.50 ~ 6.99)	0.003	2.51 (1.06 ~ 5.94)	0.037	3.1 (1.26 ~ 7.62)	0.014
*P* for trend test		0.001		0.001		0.044		0.03

Model I had no adjusted covariates

Model II adjusted for gender, age

Model III adjusted for model II plus Respiratory rate, SpO₂

Model IV adjusted for Model III plus white blood cell count, C-reactive protein, glucose, lactate

*Abbreviations*: *OR* Odds Ratio, *CI* Confidence interval

After further stratification of the hyperthermia category, severe hyperthermia remained independently associated with in-hospital mortality in the fully adjusted model (OR 5.74, 95% CI 1.55–21.20), whereas no significant association was observed for mild hyperthermia following full adjustment (Table 3). Sensitivity analyses based on the average BT measured during the first 24 hours after PICU admission yielded similar results (Supplementary Table S2).

**Table 3:** ORs for in-hospital mortality with body temperature stratified by hyperthermia groups

Variable	Model Ⅰ		Mode Ⅱ		Model Ⅲ		Model Ⅳ		
	OR (95%CI)	*P* value	OR (95%CI)	*P* value	OR (95%CI)	*P* value	OR (95%CI)	*P* value	
Normothermia (*n* = 184)	1 (Ref)		1(Ref)		1(Ref)		1(Ref)		
Mild hyperthermia (*n* = 85)	2.36 (1.01 ~ 5.49)	0.047	2.43 (1.04 ~ 5.68)	0.041	2.01 (0.79 ~ 5.13)	0.144	2.25 (0.83 ~ 6.09)	0.111	
Severe hyperthermia (*n* = 26)	6.37 (2.3 ~ 17.63)	< 0.001	6.98 (2.43 ~ 20.05)	< 0.001	5.43 (1.67 ~ 17.61)	0.005	5.74 (1.55 ~ 21.2)	0.01	

Model I had no adjusted covariates

Model II adjusted for gender, age

Model III adjusted for model II plus Respiratory rate, SpO₂

Model IV adjusted for Model III plus white blood cell count, C-reactive protein, glucose, lactate

*Abbreviations*: *OR* Odds Ratio, *CI* Confidence interval

### Results of non-linearity of BT and in-hospital mortality

**Fig. 2:**
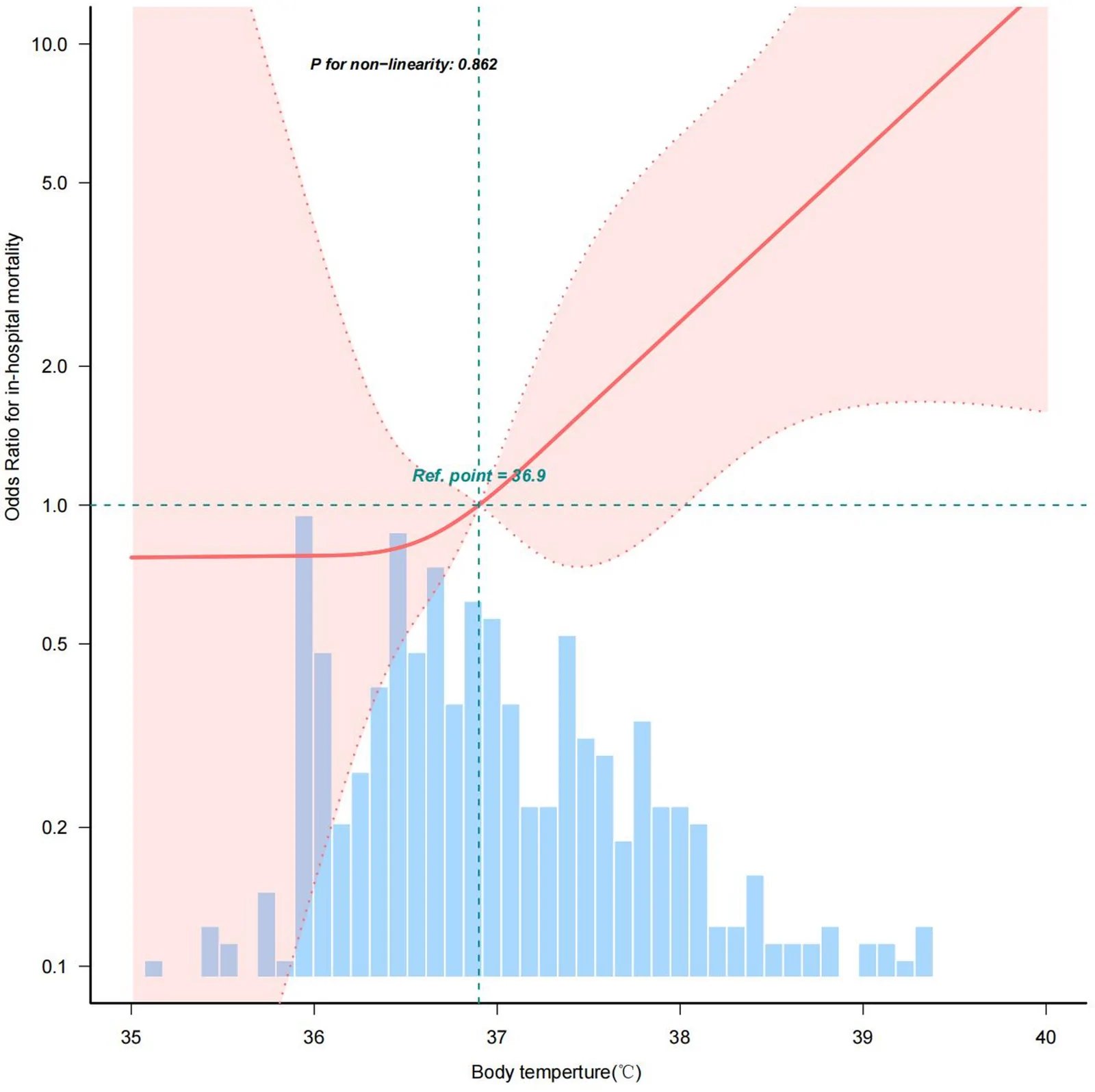
Nonlinear relationship between body temperature and in-hospital mortality. Odds Ratios (ORs) were adjusted for gender, age, Respiratory rate, SpO₂, white blood cell count, C-reactive protein, glucose, lactate

RCS analysis was performed to evaluate the potential non-linear association between BT and in-hospital mortality. As shown in Fig. 2, no significant evidence of non-linearity was observed (P for non-linearity = 0.862), indicating a predominantly linear relationship between BT and in-hospital mortality. Across the observed temperature range, the estimated risk of in-hospital death increased with higher BT levels.

### Subgroup analyses

**Fig. 3:**
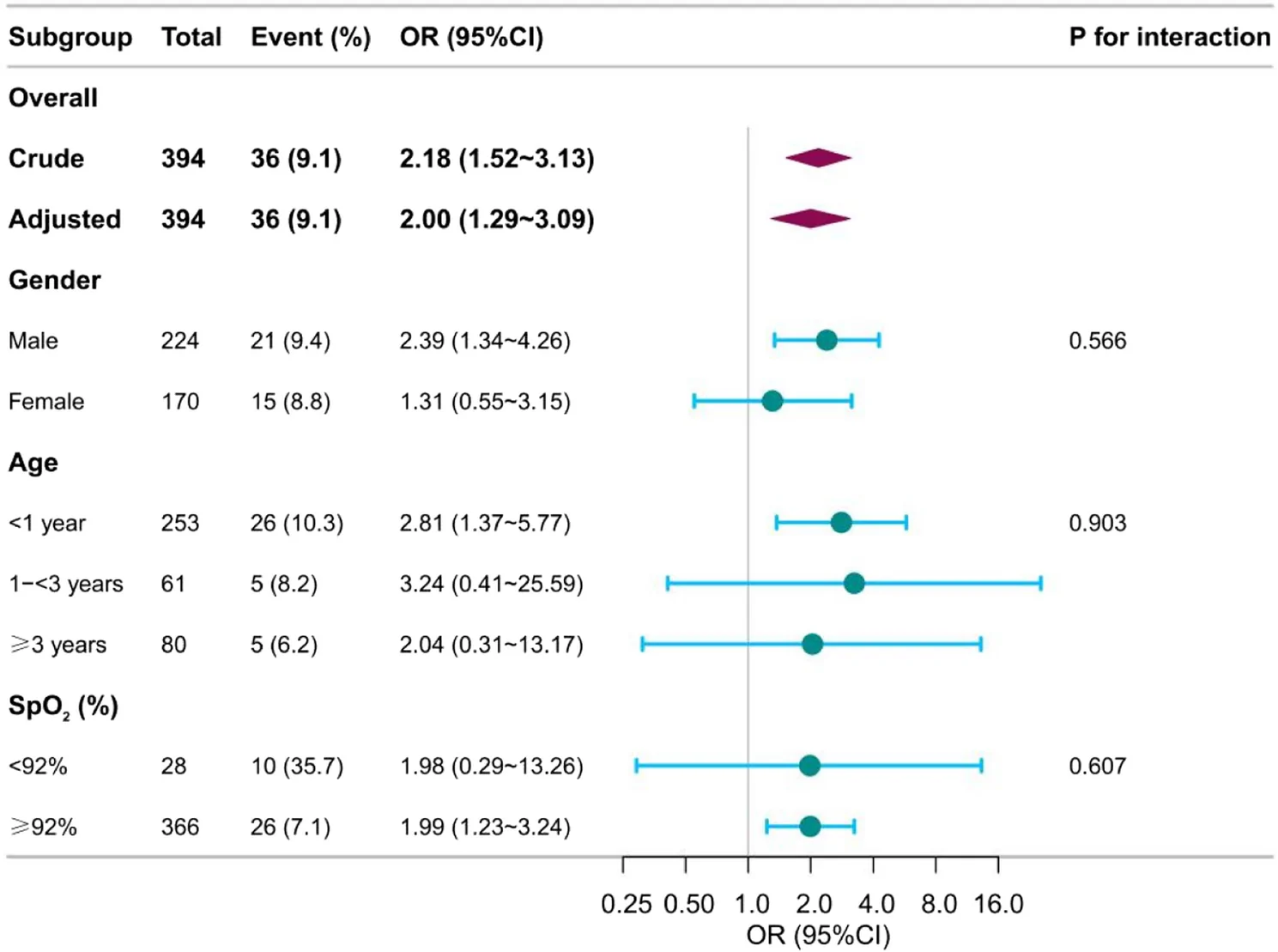
Subgroup analysis of the association between BT and in-hospital mortality. Odds Ratios (ORs) and 95% confidence intervals (CIs) are shown per 1°C increase in BT. Adjusted models were controlled for gender, age, respiratory rate, SpO₂, white blood cell count, C-reactive protein, glucose, and lactate

Figure 3 presents the results of the adjusted subgroup analyses. Elevated BT was associated with an increased risk of in-hospital mortality (OR 2.00, 95% CI 1.29–3.09). No significant interactions were observed across subgroups defined by gender, age, or SpO₂ (all P for interaction > 0.05).

### Sensitivity analysis

Analyses based on the average BT measured within the first 24 hours after PICU admission yielded findings consistent with the primary results (Supplementary Table S2). In the fully adjusted model, higher average BT was independently associated with increased in-hospital mortality (OR 1.83, 95% CI 1.12–2.99; P = 0.017). In addition, patients with hyperthermia demonstrated a significantly higher mortality risk (OR 3.13, 95% CI 1.28–7.66; P = 0.013), with a significant trend across BT categories (P for trend = 0.039). Comparisons of baseline characteristics between patients with complete and incomplete covariate data are presented in Supplementary Table S3 and showed no significant differences in demographic variables, vital signs, ICU length of stay, or in-hospital mortality. Results from the complete-case multivariable analysis (n = 215; Supplementary Table S4) showed effect estimates that were consistent in direction with the main analysis, although statistical significance was not observed. Further adjustment for vasoactive drug use yielded results that were materially unchanged from the primary analysis. Hyperthermia remained significantly associated with increased in-hospital mortality (OR 2.99, 95% CI 1.21–7.35; P = 0.017; Supplementary Table S6). Moreover, baseline characteristics were compared between patients with and without available admission BT data (Supplementary Table S5). Patients with missing BT data were younger and had a higher in-hospital mortality rate than those included in the primary analysis. Modest differences were also observed in serum calcium and phosphate levels. Nevertheless, most clinical and laboratory characteristics were broadly comparable between groups.

## Discussion

Using a large PIC database, this retrospective cohort study evaluated the association between admission BT and in-hospital mortality among critically ill children with pneumonia. The results demonstrated that higher admission BT was independently linked to an increased risk of in-hospital death. After comprehensive adjustment for potential confounders, hyperthermia was associated with an elevated risk of mortality compared with normothermia (OR 3.10, 95% CI 1.26–7.62), with a stronger association observed in cases of severe hyperthermia (OR 5.74, 95% CI 1.55–21.20). By contrast, no significant relationship was identified between hypothermia and in-hospital mortality. These findings were consistent across multiple sensitivity analyses, including analyses based on the average body temperature during the first 24 hours following PICU admission, supporting the stability of the observed associations.

Previous studies have evaluated the association between BT and adverse outcomes in critically ill and respiratory disease populations, although substantial heterogeneity exists across disease entities, age groups, and levels of illness severity. In adult ICU and infectious disease cohorts, deviations from normothermia at admission have been associated with increased mortality, with hypothermia often representing the strongest predictor of poor outcome and extreme hyperthermia also conferring excess risk [9–11, 17–20]. In adult populations, particularly among patients with acute respiratory distress syndrome (ARDS), a contrasting pattern has been reported. Schell-Chaple and colleagues observed that baseline BT, when modeled as a continuous variable, was inversely associated with 90-day mortality, with each 1 °C increase corresponding to a 15% decrease in the odds of death (OR 0.85, 95% CI 0.73–0.98, P = 0.03). These findings suggest that, in certain lung injury contexts, higher body temperature may reflect a more adaptive physiological host response [21]. However, findings from broader adult critical care and infectious disease cohorts do not support a uniformly protective role of elevated BT. In a large ICU cohort, Erkens et al. reported increased mortality among patients presenting with hypothermia at admission (OR 4.05, 95% CI 3.38–4.85), while hyperthermia was also associated with increased mortality risk [10]. Similarly, in patients with COVID-19, elevated admission or early in-hospital BT has been associated with adverse outcomes, particularly in those with high fever (BT ≥ 39-40℃) or peak BT exceeding 40℃, in whom mortality exceeded 40% [11, 18, 19].

In pediatric populations, evidence from hospitalized children with pneumonia and young infants with serious infections indicates that abnormal BT is an important marker of disease severity and mortality risk, and is therefore incorporated into pediatric severity assessment frameworks [22, 23]. In hospitalized pediatric pneumonia cohorts, Rees et al. developed the PREPARE model in 27,388 children, demonstrating a strong association between admission hypothermia and increased mortality (OR 4.80, 95% CI 3.05–7.57), with BT retained as a core continuous predictor [24]. Studies focusing on critically ill children admitted to the PICU further indicate that greater deviation of BT from the normal range is associated with higher mortality risk. Wang et al. reported that both hypothermia and hyperthermia were associated with increased 28-day mortality among children with sepsis, with risk increasing as temperature deviated further from normal values [12]. In a large pediatric intensive care cohort comprising nearly 8,000 patients, Chang et al. demonstrated that admission body temperature at both low and high extremes independently predicted 28-day mortality [13]. In addition to its utility for risk stratification, elevated body temperature may serve as an indicator of increased infectious burden and heightened systemic inflammatory activity. Christopher et al. found that among febrile children younger than five years, an axillary temperature exceeding 38.5 ℃ was strongly associated with bacteremia (OR 7.0, 95% CI 2.2–14.8), and the presence of bacteremia was, in turn, linked to a markedly higher risk of in-hospital death [25].

From a pathophysiological standpoint, changes in body temperature may exert heterogeneous and context-dependent effects. Moderate fever can, in some circumstances, limit pathogen replication and augment innate immune responses, thereby contributing to host defense mechanisms [26–30]. However, when inflammatory regulation is impaired or infectious burden is substantial, sustained or excessive hyperthermia may instead intensify pro-inflammatory signaling, including activation of NF-κB pathways, promote apoptosis of respiratory epithelial cells, and aggravate lipopolysaccharide-mediated acute lung injury, which may contribute to poorer clinical outcome [31–33]. Thermoregulatory responses may also be interpreted within the framework of energy metabolism and disease tolerance, where body temperature reflects a dynamic balance between host resistance and tolerance. In the context of severe infection, persistent hyperthermia may indicate excessive metabolic demand accompanied by depletion of physiological reserves, a state associated with increased mortality risk [26, 34, 35]. Consistent with this concept, evidence from studies evaluating antipyretic therapy suggests that fever suppression alone does not translate into a clear survival advantage, supporting the view that body temperature functions primarily as an indicator of disease severity rather than a direct therapeutic target [36].In line with these observations, our findings suggest that in critically ill children with pneumonia, abnormal body temperature–particularly markedly elevated levels–may reflect underlying disease severity and inflammatory dysregulation rather than a uniformly protective host response.

Several study limitations merit consideration. First, owing to the retrospective and observational nature of the design, causality between admission BT and mortality cannot be inferred. Although extensive adjustment for clinically relevant confounders was performed and multiple sensitivity analyses were undertaken, the possibility of unmeasured or residual confounding cannot be completely ruled out. Second, the main analyses relied on a single BT value recorded at admission, which may not adequately reflect short-term temperature variability in the early course of critical illness. Nevertheless, analyses incorporating the average BT during the first 24 hours after PICU admission produced largely consistent results, supporting the robustness of the primary findings. Similarly, although the association between hyperthermia and mortality did not reach statistical significance in the complete-case analysis, the effect estimates remained directionally consistent with those observed in the primary analysis based on multiple imputation. The loss of statistical significance was likely attributable to the substantially reduced sample size and consequent decrease in statistical power. Notably, additional adjustment for vasoactive drug use produced largely unchanged effect estimates, further supporting the robustness of the observed association. Third, information regarding antipyretic administration, physical cooling measures, emergency department management, or other active temperature management strategies before and shortly after PICU admission was not available in the database, preventing evaluation of their potential influence on admission BT and clinical outcomes. It should be noted, however, that the primary aim of this study was to assess admission BT as a prognostic indicator rather than to examine treatment effects. Furthermore, the PIC database contains data collected between 2010 and 2018. Although body temperature remains a fundamental physiological parameter whose association with mortality is unlikely to substantially change over time, the use of historical data may limit the direct applicability of our findings to current clinical practice. Another limitation is that admission BT data were unavailable for a substantial proportion of otherwise eligible patients. Comparison of patients with and without available BT data demonstrated that those with missing BT measurements were younger and had a higher in-hospital mortality rate. Therefore, selection bias cannot be completely excluded despite the overall similarity of most baseline characteristics between groups. Finally, the PIC database includes only children admitted to the PICU, representing a highly selected population of critically ill children with pneumonia. Consequently, the observed association between admission BT and in-hospital mortality should be interpreted within the context of severe pediatric pneumonia and may not be directly generalizable to children with less severe disease treated in general pediatric wards or outpatient settings. Furthermore, as the present analysis was conducted using data from a single critical care database, caution is warranted when extrapolating these findings to other clinical settings or patient populations. Future well-designed prospective, multicenter studies are needed to confirm our results and to further elucidate the impact of dynamic temperature patterns on outcomes among critically ill children with pneumonia.

## Conclusion

Admission BT was associated with in-hospital mortality among critically ill children with pneumonia, with severe hyperthermia showing the strongest association with mortality. This association persisted after multivariable adjustment, highlighting the potential relevance of early temperature abnormalities in this population. As a routinely recorded and easily accessible parameter, admission BT may contribute useful prognostic information in the PICU and aid early identification of children at increased risk, rather than serving as a direct target for therapeutic intervention. Further well-designed prospective studies across multiple centers are needed to confirm these observations and to better delineate the role of temporal temperature patterns in the clinical management of pediatric critical pneumonia.

## Supplementary Information

Supplementary Material 1.

## Data Availability

The datasets generated and/or analyzed during the current study are available from the corresponding author upon reasonable request. Please note that reanalysis of the full data for other purposes requires prior approval from the PIC Institute.

## Abbreviations

BT: Body Temperature
PIC: Pediatric Intensive Care Database
PICU: Pediatric Intensive Care Unit
ICD-10: International Classification of Diseases, 10th Revision
SQL: Structured Query Language
SpO_2_: Peripheral Oxygen Saturation
WBC: White Blood Cell Count
NEUT: Neutrophil Count
PLT: Platelet Count
HGB: Hemoglobin
CRP: C-Reactive Protein
ALB: Albumin
ALT: Alanine aminotransferase
GLU: Glucose
LDH: Lactate Dehydrogenase
K: Potassium
Ca: Calcium
P: Phosphate
OR: Odds Ratio
CI: Confidence Interval
RCS: Restricted Cubic Spline
