# Supplementary material for: Admission body temperature and in-hospital mortality in critically ill children with pneumonia: a retrospective cohort study
Source: BMC Pediatr. 2026 Jul 10;26:959. doi: 10.1186/s12887-026-07300-9 (PMC13643956; doi:10.1186/s12887-026-07300-9)
Supplement: Supplementary file 1 [file 12887_2026_7300_MOESM1_ESM.docx]

Supplementary Table S1. Univariate analysis of body temperature and in-hospital mortality

| **Variable** | **OR (95% CI)** | ***P value*** |
| --- | --- | --- |
| Gender (Female) | 0.94 (0.47~1.87) | 0.851 |
| Age(month) | 1 (1~1) | 0.771 |
| Temperature (℃) | 2.18 (1.52~3.13) | <0.001 |
| Average temperature (℃) | 2.2 (1.47~3.3) | <0.001 |
| Respiratory rate (beats/min) | 1.07 (1.04~1.09) | <0.001 |
| Heart rate (beats/min) | 1.01 (1~1.03) | 0.051 |
| SpO₂ (%) | 0.87 (0.77~0.98) | 0.028 |
| **Laboratory data** |  |  |
| WBC (×10^9^/L) | 1.04 (1~1.08) | 0.055 |
| NEUT (×10^9^/L) | 1.02 (0.96~1.08) | 0.515 |
| PLT (×10^9^/L) | 1 (1~1) | 0.75 |
| HGB（g/L） | 1.02 (1~1.04) | 0.067 |
| CRP (mg/L) | 1 (0.99~1.01) | 0.962 |
| ALB (g/L) | 1.02 (0.96~1.08) | 0.485 |
| ALT (U/L) | 1 (1~1) | 0.63 |
| LDH (U/L) | 1 (1~1) | 0.838 |
| GLU (mmol/L) | 0.74 (0.59~0.92) | 0.011 |
| Lactate (mmol/L) | 1.13 (0.97~1.31) | 0.121 |
| pH | 0.09 (0~3.15) | 0.185 |
| pCO2 (mmHg) | 1.01 (0.99~1.03) | 0.534 |
| K (mmol/L) | 1.11 (0.76~1.63) | 0.573 |
| Ca (mmol/L) | 0.01 (0~0.17) | 0.001 |
| P (mmol/L) | 1.74 (0.88~3.46) | 0.117 |

Abbreviations: SpO₂, Peripheral oxygen saturation; WBC, white blood cell count; NEUT, neutrophil count; PLT, platelet count; HGB, hemoglobin; CRP, C-reactive protein; ALB, albumin; ALT, alanine aminotransferase; LDH, lactate dehydrogenase; GLU, glucose; pCO_2_, partial pressure of carbon dioxide; K, potassium; Ca, calcium; P, phosphate. Continuous variables are described as mean ±SD or median (IQR) according to the normality of distribution.

Supplementary Table S2. Multivariate analysis of first-Day average body temperature and mortality risk

| **Variable** | **Model Ⅰ** | | **Mode Ⅱ** | | **Model Ⅲ** | | **Model Ⅳ** | |
| --- | --- | --- | --- | --- | --- | --- | --- | --- |
|  | **OR (95%CI)** | ***P value*** | **OR (95%CI)** | ***P value*** | **OR (95%CI)** | ***P value*** | **OR (95%CI)** | ***P value*** |
| **Average Temperature**  **(n = 394)** | 2.2(1.47~3.3) | <0.001 | 2.26 (1.49~3.44) | <0.001 | 1.73 (1.09~2.76) | 0.022 | 1.83 (1.12~2.99) | 0.017 |
| **Hypothermia**  **(n = 81)** | 0.8(0.25~2.54) | 0.709 | 0.79 (0.25~2.51) | 0.693 | 1.12 (0.34~3.71) | 0.852 | 1.11 (0.32~3.84) | 0.865 |
| **Normothermia**  **(n = 214)** | 1(Ref) |  | 1(Ref) |  | 1(Ref) |  | 1(Ref) |  |
| **Hyperthermia**  **(n = 99)** | 3.67(1.73~7.79) | 0.001 | 3.86 (1.79~8.33) | 0.001 | 2.64 (1.16~6.01) | 0.021 | 3.13 (1.28~7.66) | 0.013 |
| ***P* for trend test** |  | 0.001 |  | 0.001 |  | 0.076 |  | 0.039 |

Model I	had no adjusted covariates.

Model II	adjusted for gender, age.

Model III	adjusted for model II plus Respiratory rate, SpO₂.

Model IV	adjusted for Model III plus white blood cell count, C-reactive protein, glucose, lactate.

Abbreviations: OR, Odds Ratio; CI, confidence interval.

Supplementary Table S3. Baseline characteristics of patients with and without missing data.

| **Variables** | **Total** | **Missing group** | **Non-missing group** | ***P value*** |
| --- | --- | --- | --- | --- |
| **N** | 394 | 179 | 215 |  |
| Male, n (%) | 224 (56.9) | 104 (58.1) | 120 (55.8) | 0.648 |
| Age(month) | 6.8 (2.9, 22.2) | 7.0 (2.9, 29.5) | 6.8 (3.0, 17.4) | 0.337 |
| Los hospital(day) | 16.0 (8.0, 27.0) | 12.0 (4.5, 20.0) | 19.0 (12.0, 30.5) | < 0.001 |
| Los ICU (day) | 5.0 (2.0, 12.0) | 4.0 (2.0, 14.5) | 5.0 (2.0, 10.5) | 0.814 |
| In hospital mortality, n (%) | 36 (9.1) | 21 (11.7) | 15 (7) | 0.103 |
| **Vital signs** |  |  |  |  |
| Respiratory rate (beats/min) | 39.3 ± 12.3 | 39.3 ± 12.3 | 39.3 ± 12.4 | 0.998 |
| Heart rate (beats/min) | 138.1± 26.4 | 140.1 ± 29.0 | 136.8 ± 24.5 | 0.241 |
| SpO₂ (%) | 97.3 ± 3.0 | 97.4 ± 2.3 | 96.3 ± 3.1 | 0.84 |
| **Laboratory data** |  |  |  |  |
| WBC (×10^9^/L) | 9.0 (5.9, 13.9) | 9.9 (6.5, 14.4) | 8.2 (5.6, 13.7) | 0.039 |
| NEUT (×10^9^/L) | 5.0 (2.9, 9.6) | 5.8 (3.3, 10.4) | 4.8 (2.6, 8.8) | 0.044 |
| PLT (×10^9^/L) | 311.7 ± 168.2 | 325.6 ± 164.6 | 301.0 ± 170.6 | 0.158 |
| HGB（g/L） | 104.7 ± 18.8 | 106.3 ± 19.8 | 103.4 ± 18.0 | 0.14 |
| CRP (mg/L) | 5.0 (2.0, 22.6) | 4.0 (1.9, 15.7) | 6.0 (2.0, 33.5) | 0.259 |
| ALB (g/L) | 37.2 ± 6.1 | 37.4 ± 6.3 | 37.2 ± 5.9 | 0.748 |
| ALT (U/L) | 25.0 (17.0, 40.0) | 26.5 (16.0, 45.2) | 23.0 (17.0, 35.0) | 0.278 |
| LDH (U/L) | 396.0 (298.5, 565.0) | 393.0 (295.5, 611) | 397.0 (300.0, 550.0) | 0.945 |
| GLU (mmol/L) | 7.5 ± 3.0 | 6.9 ± 2.8 | 7.9 ± 3.0 | 0.002 |
| Lactate (mmol/L) | 1.7 (1.2, 2.5) | 1.8 (1.2, 2.9) | 1.7 (1.2, 2.4) | 0.318 |
| pH | 7.4 ± 0.1 | 7.4 ± 0.1 | 7.4 ± 0.1 | 0.074 |
| pCO2 (mmHg) | 45.6 ± 16.3 | 47.0 ± 18.5 | 44.4 ± 14.3 | 0.128 |
| K (mmol/L) | 4.0 ± 0.8 | 4.0 ± 1.0 | 3.9 ± 0.7 | 0.028 |
| Ca (mmol/L) | 1.2 ± 0.1 | 1.2 ± 0.1 | 1.2 ± 0.1 | 0.471 |
| P (mmol/L) | 1.6 ± 0.5 | 1.7 ± 0.6 | 1.5 ± 0.4 | 0.02 |

Supplementary Table S4. Multivariable logistic regression for in-hospital mortality after complete-case analysis (excluding all records with missing values).

| **Variable** | **Model Ⅰ** | | **Mode Ⅱ** | | **Model Ⅲ** | | **Model Ⅳ** | |
| --- | --- | --- | --- | --- | --- | --- | --- | --- |
|  | **OR (95%CI)** | ***P value*** | **OR (95%CI)** | ***P value*** | **OR (95%CI)** | ***P value*** | **OR (95%CI)** | ***P value*** |
| **Temperature**  **(n = 215)** | 1.99 (1.1~3.58) | 0.022 | 2.1 (1.14~3.85) | 0.017 | 1.7 (0.88~3.28) | 0.114 | 1.98 (0.94~4.15) | 0.07 |
| **Hypothermia**  **(n = 59)** | 0.23 (0.03~1.91) | 0.173 | 0.22 (0.03~1.86) | 0.165 | 0.39 (0.04~3.43) | 0.395 | 0.44 (0.05~4.16) | 0.476 |
| **Normothermia**  **(n = 100)** | 1 (Ref) |  | 1(Ref) |  | 1(Ref) |  | 1(Ref) |  |
| **Hyperthermia**  **(n = 56)** | 1.9 (0.63~5.72) | 0.255 | 2.03 (0.66~6.2) | 0.216 | 1.84 (0.56~6.03) | 0.317 | 2.53 (0.67~9.52) | 0.169 |
| ***P* for trend test** |  | 0.028 |  | 0.023 |  | 0.123 |  | 0.083 |

Model I	had no adjusted covariates.

Model II	adjusted for gender, age.

Model III	adjusted for model II plus Respiratory rate, SpO₂.

Model IV	adjusted for Model III plus white blood cell count, C-reactive protein, glucose, lactate.

Abbreviations: OR, Odds Ratio; CI, confidence interval.

Supplementary Table S5. Comparison of baseline characteristics between patients with and without admission BT data

| **Variables** | **Total** | **BT Available group** | **BT Missing group** | ***P value*** |
| --- | --- | --- | --- | --- |
| **N** | 965 | 394 | 571 |  |
| Male, n (%) | 575 (59.6) | 224 (56.9) | 351 (61.5) | 0.151 |
| Age(month) | 6.3(2.6, 19.5) | 6.8 (2.9, 22.2) | 5.4 (2.5, 17.7) | 0.041 |
| Los hospital(day) | 13.0 (6.0, 23.0) | 16.0 (8.0, 27.0) | 11.0 (5.0, 21.0) | < 0.001 |
| Los ICU (day) | 6.0 (3.0, 13.0) | 5.0 (2.0, 12.0) | 7.0 (3.0, 14.0) | 0.068 |
| In hospital mortality, n (%) | 137 (14.2) | 36 (9.1) | 101 (17.7) | < 0.001 |
| **Vital signs** |  |  |  |  |
| Respiratory rate (beats/min) | 36.0 (30.0, 48.0) | 36.0 (30.0, 48.0) | 36.0 (30.0, 46.0) | 0.698 |
| Heart rate (beats/min) | 139.4 ± 26.4 | 138.1 ± 26.4 | 141.7 ± 26.3 | 0.121 |
| **Laboratory data** |  |  |  |  |
| WBC (×10^9^/L) | 8.8 (5.8, 13.5) | 9.0 (5.9, 13.9) | 8.7 (5.8, 12.5) | 0.153 |
| NEUT (×10^9^/L) | 4.8 (2.7, 8.6) | 5.0 (2.9, 9.6) | 4.7 (2.7, 7.7) | 0.087 |
| PLT (×10^9^/L) | 299.3 ± 168.7 | 311.7 ± 168.2 | 288.8 ± 168.5 | 0.052 |
| HGB（g/L） | 104.0 ± 20.2 | 104.7 ± 18.8 | 103.5 ± 21.4 | 0.401 |
| CRP (mg/L) | 6.0 (2.0, 25.0) | 5.0 (2.0, 22.6) | 6.0 (3.0, 26.0) | 0.068 |
| ALB (g/L) | 37.2 ± 5.9 | 37.2 ± 6.1 | 37.1 ± 5.8 | 0.696 |
| ALT (U/L) | 24.0 (16.8, 40.0) | 25.0 (17.0, 40.0) | 24.0 (16.0, 40.0) | 0.773 |
| LDH (U/L) | 402.0 (301.0, 577.0) | 396.0 (298.5, 565.0) | 405.5 (304.2, 601.0) | 0.446 |
| GLU (mmol/L) | 7.6 ± 3.4 | 7.5 ± 3.0 | 7.6 ± 3.7 | 0.484 |
| Lactate (mmol/L) | 1.7 (1.2, 2.6) | 1.7 (1.2, 2.5) | 1.7 (1.2, 2.6) | 0.965 |
| pH | 7.4 ± 0.1 | 7.4 ± 0.1 | 7.4 ± 0.1 | 0.089 |
| pCO2 (mmHg) | 45.6 ± 17.2 | 45.6 ± 16.3 | 45.7 ± 18.0 | 0.916 |
| K (mmol/L) | 4.0 ± 0.8 | 4.0 ± 0.8 | 3.9 ± 0.7 | 0.280 |
| Ca (mmol/L) | 1.2 ± 0.1 | 1.2 ± 0.1 | 1.2 ± 0.1 | 0.031 |
| P (mmol/L) | 1.7 ± 0.6 | 1.6 ± 0.5 | 1.7 ± 0.6 | 0.005 |

Supplementary Table S6. Sensitivity analysis examining the association between admission body temperature and in-hospital mortality after additional adjustment for vasoactive drug use.

| **Variable** | **Temperature**  **(n = 394)** | | **Hypothermia**  **(n = 99)** | | **Normothermia**  **(n = 184)** | | **Hyperthermia**  **(n = 111)** | |
| --- | --- | --- | --- | --- | --- | --- | --- | --- |
|  | **OR (95%CI)** | **P value** | **0R (95%CI)** | **P value** |  | **P value** | **OR (95%CI)** | **P value** |
| Model IV+ Vasoactive drug | 1.99 (1.27~3.13) | 0.003 | 0.98 (0.28~3.5) | 0.977 | Reference | - | 2.99 (1.21~7.35) | 0.017 |

Model IV adjusted for gender, age, respiratory rate, SpO_2_, white blood cell count, C-reactive protein, glucose, and lactate.

Abbreviations: OR, odds ratio; CI, confidence interval.
